# C-reactive Protein Levels in Patients With Autoimmune Hypothyroidism Before and After Levothyroxine Treatment

**DOI:** 10.7759/cureus.50848

**Published:** 2023-12-20

**Authors:** Stela Vudu, Andrew Behnke

**Affiliations:** 1 Diabetes and Endocrinology, Nicolae Testemițanu State University of Medicine and Pharmacy, Chisinau, MDA; 2 Diabetes and Endocrinology, Virginia Tech Carilion School of Medicine, Roanoke, USA

**Keywords:** hashimoto's hypothyroidism, sub-clinical hypothyroidism, beneficial effects of levothyroxine, c-reactive proteins, autoimmune hypothyroidism

## Abstract

Background: Hypothyroidism is one of the most common endocrine disorders. Most patients with hypothyroidism have autoimmune thyroiditis (Hashimoto's), characterized by elevated concentrations of anti-thyroperoxidase (ATPO) antibodies. Both overt hypothyroidism (OH) and subclinical hypothyroidism (SH) have been associated with cardiovascular risk factors, including markers of inflammation. High-sensitivity C-reactive protein (hs-CRP) is a veridical marker of systemic inflammation. Even a minor increase in hs-CRP is considered a cardiovascular risk; therefore, evidence of a beneficial effect of levothyroxine treatment on hs-CRP could be an argument in favor of therapy for SH.

Aim: To assess hs-CRP levels in patients with hypothyroidism and evaluate levothyroxine treatment's effect on hs-CRP.

Study design: This is a cohort study in which patients with hypothyroidism were evaluated before and after treatment with levothyroxine.

Methods: 37 patients (17 with OH and 20 with SH) and 38 healthy controls were included in the study. hs-CRP was measured at the baseline visit, then after 2 and 4 months of levothyroxine therapy at a dose necessary to achieve euthyroidism as evidenced by a normal level of thyroid-stimulating hormone (TSH).

Results: hs-CRP was significantly increased in OH (p < 0.001) and SH (p = 0.001) at baseline as compared to controls. hs-CRP significantly decreased in SH (2.2±1.6 mg/L at baseline visit, 1.4±1.1 mg/L after 2 months of levothyroxine treatment, P = 0.017) and tended to decrease in OH (2.3±1.6 mg/L at baseline visit, 1.6±1.1 mg/L after 4 months of levothyroxine treatment, P = 0.067*).

Conclusions: Patients with hypothyroidism have increased hs-CRP levels compared to a healthy control group and, thereby, a moderately increased cardiovascular risk. Achievement of euthyroidism by levothyroxine treatment decreased the levels of hs-CRP in patients with hypothyroidism.

## Introduction

Hypothyroidism is one of the most common endocrine disorders. In many cases, the diagnosis is clear and confirmed with an elevated Thyroid Stimulating Hormone ( TSH) and low free thyroxine levels. Subclinical hypothyroidism (SH) is defined by elevated TSH and free thyroxine levels within the reference range, usually between 0.4 to 4.0 mU/L. This diagnosis is established primarily based on laboratory tests; the clinical picture found in these patients is non-specific. The prevalence of SH ranges from 4 to 15%, being higher with increasing age and in women [1]. Most patients with SH have autoimmune thyroiditis (Hashimoto's), characterized by elevated concentrations of anti-thyroperoxidase (anti-TPO) antibodies [2].

The consequences of SH are numerous: progression to overt hypothyroidism, reproductive disorders, and, in some studies, increased body weight [3,4]. SH may be associated with an increased risk of cardiovascular disease, including ischemic heart disease, heart failure, and hypercholesterolemia [2,5,6]. SH has also been associated with cardiovascular risk factors, including markers of inflammation [7]. C-reactive protein (CRP) is a veridical marker of systemic inflammation. High-sensitivity CRP (hs-CRP) is more accurate than standard CRP when assessing baseline (normal) values and can be used to measure chronic inflammation.

An important clinical question is whether patients with SH should be treated with levothyroxine. In general, data are contradictory and depend on several factors. These include the level of TSH, the titer of anti-thyroid antibodies, and age [8]. Elevated hs-CRP is not currently used as a factor for levothyroxine treatment. As even a minor increase in CRP is considered a cardiovascular risk [9], evidence of the beneficial effect of levothyroxine treatment on hs-CRP could be an argument in favor of therapy.

Previous studies have shown different results on CRP levels in patients with hypothyroidism. M. Christ-Crain et al. were the first to demonstrate that CRP increases hypothyroidism, but levothyroxine treatment did not ameliorate the results [10]. Kvetny J et al. also showed that SH is associated with increased CRP [7]. We aimed to assess the level of hs-CRP in patients with hypothyroidism and to evaluate the (potential) effect of levothyroxine treatment on hs-CRP.

This article was previously presented as a poster at the 2023 European Society of Endocrinology (ESE) Annual Scientific Meeting on May 13, 2023.

## Materials and methods

Patients who were referred to an endocrinologist with hypothyroidism symptoms were asked to participate in the study. The patients requested the consultation of the endocrinologist due to the symptoms of fatigue, lack of energy, constipation, dryness of the skin, or irregular menstrual cycle. Thirty-seven patients with untreated autoimmune hypothyroidism (17 - with overt hypothyroidism (OH) and 20 - with SH) and 38 subjects in the control group were included in the study. Four of the 17 with OH and one of the 20 with SH were male. The study protocol was approved by the Ethics Committee of the "Nicolae Testemițanu" State University of Medicine and Pharmacy, Chisinau, Republic of Moldova. Inclusion criteria were age between 18 and 65 years and a diagnosis of OH or SH. The patients requested the consultation of the endocrinologist due to the symptoms of fatigue, lack of energy, constipation, dryness of the skin, or irregular menstrual cycle. Overt hypothyroidism was defined as TSH greater than 4 μIU/mL and free T4 below reference values. Subclinical hypothyroidism was defined as TSH greater than 4 μIU/mL and free T4 within reference values (0.69 - 1.7 ng/dL). In SH, thyroid dysfunction was confirmed by repeated TSH testing at least 1 month apart.

Subjects with diabetes, class II and III obesity, pregnancy, lactation, history of respiratory or cardiovascular diseases, systemic diseases, or administration of certain drugs (levothyroxine, methimazole, amiodarone, statins, vitamins) were excluded. TSH, free T4, total cholesterol (TC), HDL-C, LDL-C, creatinine, creatine phosphokinase (CK), and hs-CRP were assessed in all participants. The same laboratory analyses were performed after 2 and 4 months of levothyroxine treatment. Complete blood count and anti-thyroid antibodies were performed only at the pre-treatment stage. In patients with overt hypothyroidism, the initial dose of levothyroxine was 1.6 mcg/kg/day; in subclinical hypothyroidism, it was 0.7 mcg/kg/day. After 2 months, the dose was adjusted, if necessary, by 12.5, 25, or 50 mcg/day to obtain a TSH in the reference range. Physical examination and laboratory investigations were performed on all patients. Body mass index (BMI) was calculated using the formula based on body weight and height.

Thyroid function tests were evaluated by the direct chemiluminescence method, a quantitative test (Immulite 2000, Siemens Healthcare Diagnostics, USA). TSH reference values are 0.4 - 4 µIU/mL, for free T4: 0.69 - 1.7 ng/dL, anti-TPO < 10 IU/mL, anti-TG (ATG) < 20 IU/mL. TC was assessed by the colorimetric method, LDL-c, and HDL-c - by spectrophotometry. TC reference values are < 5.2 mmol/L, for LDL-c: < 2.6 mmol/L, for HDL-c: >= 1.55 mmol/L. The kinetic method assessed Creatinine and creatine phosphokinase with reference values: 45.0 - 84.0 µmol/L and < 145.0 U/L, respectively. Hs-CRP was assessed by the latex immunoturbidimetric method. Reference value: < 3.33 mg/L. The blood count was performed by the flow cytometry method.

Quantitative variables were analyzed using parametric tests (one-way ANOVA) and non-parametric tests (Kruskal Wallis). The effect of treatment was assessed by paired t-test and Wilcoxon signed Rank test. Proportions were analyzed using Pearson Chi-Square tests with Bonferroni correction for multiple comparisons.

## Results

Thirty-seven patients with autoimmune hypothyroidism (17 with OH and 20 with SH) and 38 age- and sex-matched healthy controls were included in the study. Anthropometric and laboratory data at baseline are presented in Table 1.

**Table 1:** Anthropometric data, thyroid hormones profile, laboratory data in patients with hypothyroidism and healthy controls. Data are mean ± Standard deviation. p<0.05** -  for comparison between OH and healthy controls, p<0.05‡ - for comparison between SH and healthy controls, p<0.0001* - for comparison between OH and healthy controls, p=0.001‡ for comparison between SH and healthy controls. OH -Overt hypothyroidism; SH-Subclinical hypothyroidism; BMI-Body Mass Index; TSH- Thyroid Stimulating Hormone; fT4- Free Thyroxine; fT3- Free Triiodothyronine; ATPO- Thyroperoxidase Antibody; ATG- Thyroglobulin Antibody; TC- Total Cholesterol; LDL-c - Low Density Lipoprotein; HDL-c - High Density Lipoprotein; CK- Creatinine Kinase; hs-CRP -Highly Sensitive C- Reactive Protein

	OH	SH	Healthy	P
Age (years)	43.5 ± 11.1	39.1± 13.4	40.1 ± 10.1	
BMI (Kg/M2)	28.4 ±4	25 ± 4.1	24.8 ± 4.3	
TSH (uIU/ml)	104.5 ± 100	6.7 ± 2.4	2 +± 0.7	<0.001*
fT4 (ng/dl)	0.4 ± 0.2	0.9 ± 0.1		
fT3 (pg/ml)	2.2 ± 1.2	3.2 ± 0.5		
ATPO (IU/ml)	629.2 ± 398.3	517.9 ± 369.3	21 ± 35.9	<0.001*‡
ATG (IU/ml)	92.4 ± 116.7	271.7 ± 66.7		
TC (mmol/L)	7.4 ± 2.3	5.0 ± 1.1	5.4 ± 0.2	
LDL-c (mmol/l)	4.9 ± 1.8	3.1 ± 0.8	3.2± 0.7	
HDL-c (mmol/l	1.6 ± 0.7	1.5 ± 0.3	1.7+-0.3	
Creatinine (μmol/l)	80.1 ± 19.8	57.5± 11.9	57.6± 8.4	
CK (U/L)	866.8 ± 117.9	118.9 ± 87.6		
hs-CRP (mg/L)	2.3± 1.6	2..2 ± 1.6	0.9 ± 0.4	<0.001* (0.001‡)

Patients with OH had higher TSH, anti-TPO, TC, LDL-c, serum creatinine, and hs-CRP compared to healthy controls. Patients with SH had higher TSH, anti-TPO, and hs-CRP and lower HDL-c compared to healthy controls. TC, LDL-c, and serum creatinine were not statistically different from healthy controls. Levothyroxine treatment did not change the level of TC, HDL-c, LDL-c, CK, and serum creatinine in patients with SH but decreased these biomarkers in patients with OH (p<0,005) (Table 2).

**Table 2:** Laboratory data before and after levothyroxine treatment in SH SH- Subclinical Hypothyroidism; TSH- Thyroid Stimulating Hormone; TC- Total Cholesterol; LDL-c - Low-Density Lipoprotein; HDL-c - High-Density Lipoprotein; CK- Creatinine Kinase; hs-CRP- Highly Sensitive C- Reactive Protein

Parameter	Before Treatment	After treatment (p, Anova test)
TSH (µIU/mL)	6.7±2.4	3.7±2.5 (p<0,001)
TC (mmol/L)	5±1.1	5.2±1.2 (n.s.)
LDL-c (mmol/L)	3.1±0.8	3.3±1 (n.s.)
HDL-c (mmol/L)	1.5±0.3	1.5±.,3 (n.s.)
Creatinine (μmol/l)	57.4±10.2	57.5±11.9 (n.s.)
CK (U/L)	118.9±87.6	103.6±59.3 (n.s.)
hs-CRP (mg/L)	2.2±1.6	1.4 ± 1.1 (p=0.017)

Hs-CRP significantly decreased in SH: 2.2±1.6 mg/L at baseline visit, 1.4±1.1 mg/L after two months of treatment, p=0.017 (Table 2) and tended to decrease in OH (2.3±1.6 mg/L at baseline visit, 1.6±1.1 mg/L after four months of treatment, p=0.067* (Table 3).

**Table 3:** Laboratory data before and after levothyroxine treatment in OH OH- Overt hypothyroidism; TSH- Thyroid Stimulating Hormone; TC- Total Cholesterol; LDL-c - Low-Density Lipoprotein; HDL-c - High-Density Lipoprotein; CK- Creatinine Kinase; hs-CRP- Highly Sensitive C- Reactive Protein

Parameter	Before Treatment	After treatment (p, Anova test)
TSH (µIU/mL)	104.5± 100	8.1± 14.4 (p<0.001)
TC (mmol/L)	7.4± 2.3	5.5±1.6 (p<0.003)
LDL-c (mmol/L)	4.9± 1.8	3.4± 1.3 (p=0.013)
HDL-c (mmol/L)	1.6± 0.7	1.5± 0.6 (n.s.)
Creatinine (μmol/l)	80.1± 19.8	62.9± 13.1 (p<0.001)
CK (U/L)	866.8 ± 171.9	92.8± 46.3 (p<0.014)
hs-CRP (mg/L)	2.3± 1.6	1.6 ± 1.1 (p=0.067)

The mean hs-CRP in the control group was 0.9±0.4 mg/L. A statistically significant difference was reached in hs-CRP median values after levothyroxine treatment in both overt (p=0.022) and SH (p=0.013) (Table 4).

**Table 4:** Median of hs-CRP in patients with OH and SH before and after treatment OH: Overt hypothyroidism; SH: Subclinical hypothyroidism

	Before Treatment	After Treatment	p (Wilcoxon test)
OH	1.8	1.3	0.022
SH	1.6	1.1	0.013

**Figure 1:**
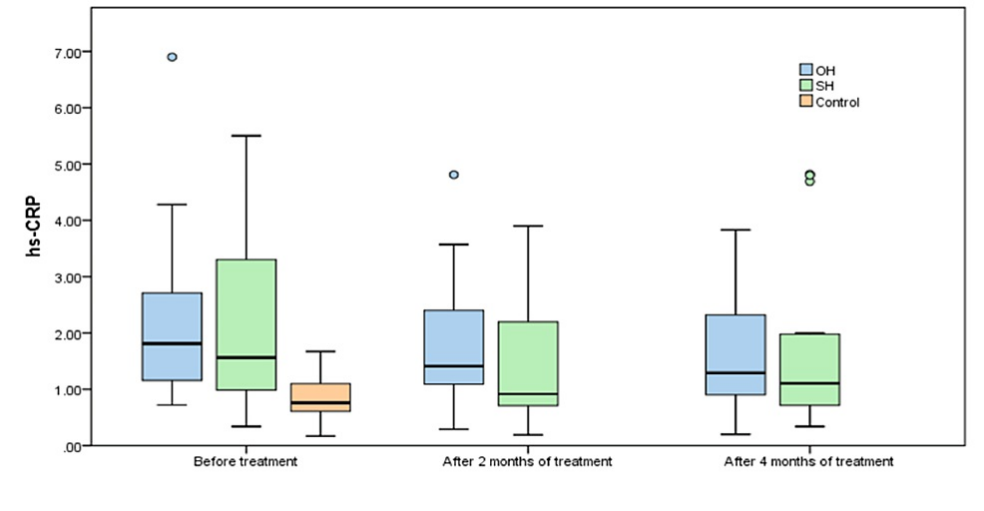
Box plot of hs-CRP distribution in OH, SH versus healthy controls before and after treatment with levothyroxine OH: overt hypothyroidism; SH: subclinical hypothyroidism

Only 17.6% of patients with OH and 30% of those with SH had a hs-CRP < 1 mg/L at baseline visit, as compared to the control group, where 71.1% of persons were at low cardiovascular risk. After 4 months of treatment, 41.2% and 40% of patients with OH and SH, respectively, decreased their hs-CRP < 1 mg/L (Fig 1).

## Discussion

This study reveals that patients with OH had higher TC and LDL-c, and patients with SH had lower HDL-c levels compared to healthy controls. Whereas OH is associated with changes in lipid profile and increased total and LDL-cholesterol, most patients with SH have normal lipid concentrations, as presented here. Although a few studies have shown that some patients with SH have slightly high serum total and LDL cholesterol, this was not seen in this study [11,12].

The results also revealed that persons with autoimmune hypothyroidism had increased hs-CRP compared to healthy subjects. In addition, hs-CRP decreased after two months of treatment with levothyroxine in patients with SH and tended to decrease after four months of treatment in OH, indicating that more time is probably needed with a more severe thyroid failure.

Elevated hs-CRP levels are markers of systemic inflammation and increased cardiovascular risk [13]. Hypothyroidism (both overt and subclinical) may be associated with increased cardiovascular risk, but the studies have varying results [14-16]. A meta-analysis by Tellechea M.L. reported elevated CRP in patients with hypothyroidism [17]. Overall, CRP decreased in OH due to levothyroxine treatment, suggesting that CRP surveillance, in addition to TSH, should be considered [13]. 

Another small study of 30 patients with SH showed a beneficial effect of levothyroxine treatment on arterial stiffness but not hs-CRP. In addition to being limited by size, some did respond, and some did not [18]. Although Won-Young Lee et al. found that CRP levels were unaffected by the degree of thyroid dysfunction, that study only looked at overall levels and did not follow subjects before and after treatment [19].

SH is a disease diagnosed solely on biochemical changes (increased TSH), and treatment decisions may be debatable. Some studies show no cardiovascular advantages of levothyroxine treatment [20,21], while others suggest a benefit [22,23]; thereby, more trials are needed to estimate the role of levothyroxine in decreasing the cardiovascular risk in subclinical hypothyroidism; until then, an individual approach is well-advised. This clinical use of hs-CRP may be another tool to use in the decision to treat patients with SH.

A strength of this study is that it was a prospective study in which patients with hypothyroidism were evaluated before and after treatment with levothyroxine. The patients were homogenous within the two distinct groups (with OH and SH, respectively). A small sample size limited the power of the study, yet it was still able to meet statistical significance.

## Conclusions

In this study, patients with both overt and subclinical hypothyroidism had increased CRP levels compared to a healthy control group and, thereby, an increased cardiovascular risk. Achievement of euthyroidism by thyroid hormone replacement decreased the levels of hs-CRP. Although SH should be treated on a case-by-case approach, using hs-CRP may aid in the decision to treat thyroid hormone. Specifically, if a patient has cardiovascular disease or risk factors, measuring hs-CRP before and after treatment with thyroid hormone may help in clinical management.
